# Supplementary material for: The structure balance of gene-gene networks beyond pairwise interactions
Source: PLoS One. 2022 Mar 30;17(3):e0258596. doi: 10.1371/journal.pone.0258596 (PMC8967046; doi:10.1371/journal.pone.0258596)
Supplement: S1 File — (PDF) [file pone.0258596.s001.pdf]

All genes with the highest degree of balance classified in terms of biological processes annotating are represented in the following table. The gene ontology of them has been explored from https://www.yeastgenome.org/ through this path: function> gene ontology> GO Slim Mapper.

GO Slim processes	Allele name
DNA recombination (GO:0006310)	CSM2,CSM4,DMC1,EXO1,HOP2,IRC16,IRC20,IRC8,MLH1,MSC3,MSC7,MSH5,NDJ1,PCH2,PDR10,RDH54,RIM4,WSS1,YKU70,YKU80,ZIP1,ZIP2
DNA repair (GO:0006281)	APN1,CSM2,DIN7,EAF6,EXO1,IRC20,MLH1,NUP120,PDR10,RAD30,RAD33,RDH54,REV1,REV3,REV7,TEL1,TPK1,UNG1,WSS1,YKU70,YKU80
DNA replication (GO:0006260)	BMH2,FKH1,NPL4,RIM4,SAK1
DNA-templated transcription, elongation (GO:0006354)	FKH1,ISW1,JHD1,STB5,TYE7
DNA-templated transcription, initiation (GO:0006352)	ISW1,MED2,ROX3
DNA-templated transcription, termination (GO:0006353)	FKH1,ISW1
Golgi vesicle transport (GO:0048193)	AGE1,AVL9,CSR1,ELO3,EMP47,ERP4,ERP6,GGA1,GGA2,GYL1,GYP5,KTR6,SLY41,SMY2,SRO77,SVP26,TCA17,TIP20,VTH1
RNA catabolic process (GO:0006401)	AIR2,CUE2,DBR1,DCS2,DXO1,HEK2,IGO2,JSN1,NGL3,NGR1,NPL4,PUB1,PUF2,RNH1,STO1,TIS11,TPA1,TRF5,WHI4
RNA splicing (GO:0008380)	CUS2,DBR1,STO1
amino acid transport (GO:0006865)	AGC1,AQR1,ATG22,AVT1,MUP3,RTC2,TAT1,TAT2,VBA2,YCT1,YHC3,YMC1,YPQ2
carbohydrate metabolic process (GO:0005975)	BMH1,BMH2,DAK1,DAK2,DOG2,ENO1,ENO2,GAC1,GAL1,GAL80,GLK1,GPP2,GUT1,HXK1,INM2,MAL13,MDH2,PCK1,PFK27,PGM2,PHO13,PIG2,PSK2,PYC1,PYC2,RBS1,SAK1,SDH7,SGA1,SKN1,STD1,SUC2,TYE7,XKS1,YIG1,YMR099C
carbohydrate transport (GO:0008643)	GLK1,GUP2,HXK1,HXT10,HXT4,HXT5
cell budding (GO:0007114)	AXL1,BUD2,BUD4,DCW1,PPH22,RAX1,RAX2,TGL3,TGL4
cell morphogenesis (GO:0000902)	FUS1,FUS2,KEL1,RSP5,YCK2
cell wall organization or biogenesis (GO:0071554)	BIT61,BMH1,BMH2,CCW14,CDA1,CDA2,CRH1,CRR1,CRZ1,CWP2,DCW1,DIT1,DIT2,FKS3,FLC1,GAS4,GIP1,HLR1,KTR6,KTR7,LDS2,MHP1,MKC7,MKK2,MSG5,NCE102,OSW2,PTP3,QDR3,SDP1,SED1,SIM1,SKN1,SMK1,SPO73,SPS2,SSP1,SVP26,TAX4,TOR1,UTR2,YEA4,YEH2,YLR194C,YPS1,YPS3
cellular amino acid metabolic process (GO:0006520)	ADH1,AGX1,ARO10,ASP1,BNA6,CAR2,DTD1,FMP32,FPR1,HFD1,IDP1,LEU1,LEU9,MAE1,MET17,MET6,PDC1,PDC6,PUT1,SAM1,SAM2,SDL1,TRP2,UGA1,UGA2,UTR4
cellular ion homeostasis (GO:0006873)	ARN2,COA6,COT1,GRX3,GRX4,HAL5,HRK1,ISU1,IZH4,MMT2,MSC2,PGM2,PIC2,PPZ1,PPZ2,PTK2,STV1,TIS11,VHS3,YDR089W,YHC3,YVC1,ZRC1
cellular respiration (GO:0045333)	GDS1,QCR10,RNR3,RSF1,SHH4,TAZ1,YER156C,YMR31
cellular response to DNA damage stimulus (GO:0006974)	BMH1,BMH2,CKA1,DDR2,TEL1,TOR1,UBP12,WSS1,YNK1,YPR015C

chromatin organization (GO:0006325)	ESC1,ESC8,FKH1,FPR1,FPR3,FPR4,HFI1,ISW1,MSN2,NUP120,PNC1,RSP5,YKU70,YKU80,ZDS2
chromosome segregation (GO:0007059)	AMN1,CSM2,CSM4,DMC1,ESC8,FIN1,GAC1,GIP4,HOP1,HOP2,ISW1,MAM1,NDJ1,RAD30,RDH54,RED1,SPO11,SPO13,ZIP1,ZIP2
conjugation (GO:0000746)	AFB1,AGA2,AXL1,CSN9,DNF1,DNF3,FUS1,FUS2,KEL1,KSS1,MF(ALPHA)2,MSG5,OSH3,POG1,PRR2,PTP3,RRI1,RRI2,RSP5,YPS1
cytokinesis (GO:0000910)	AXL1,BNR1,BUD2,BUD4,BUD8,DMA2,KEL1,KSS1,MLC2,RAX1,RAX2,VHS2
cytoplasmic translation (GO:0002181)	PSK2,RPL21A,RPL22B,RPL34B,RPL35A,RPS26A,RPS27B,RPS4A,RRT2,SLH1,TIF3,TMA108,TMA19,TMA46,TPK2,TPK3
cytoskeleton organization (GO:0007010)	ACF2,AVO2,BEM2,BIT61,BNR1,BUD4,DMA2,FIN1,GRX3,GRX4,HSP42,KEL1,LIA1,LSB1,MHP1,MLC2,NPL4,PIN3,PPH22,RGA2,RSP5,SCP1,SLM1,TDA2,VHS2,YBP2,YGL015C
endocytosis (GO:0006897)	ALY2,BRE4,BUL1,DNF1,HAL5,LSP1,OSH2,OSH3,OSH6,ROY1,RSP5,SDS24,SUR7,SVL3,SWH1,UBX3,YAP1801,YCK2
histone modification (GO:0016570)	ACS1,EAF6,FPR4,GRX3,GRX4,HFI1,HOS3,JHD1,SET5,SNT2,TEL1,UBX3,XBP1
ion transport (GO:0006811)	AGC1,AGP2,ALR2,AQR1,ARN1,ARN2,ATO2,ATO3,ATR1,AVT1,BSD2,BUL1,COT1,DUR3,ECM7,FAA4,FET4,FET5,FLC1,FLC3,GEM1,GEX2,GIT1,JEN1,MEP3,MME1,MRS4,MSC2,MUP3,NRT1,PHO84,PHO89,PHO91,PIC2,POR2,PTK1,PTK2,SAL1,SIA1,SSU1,SUL2,TAT1,TAT2,THI7,THI72,TPO4,YCF1,YCT1,YEA4,YHC3,YHL008C,YMC1,YMD8,YVC1,ZRG17
lipid metabolic process (GO:0006629)	ALE1,ATF2,ATG15,CLD1,CPT1,CSR1,DPL1,ECI1,ECT1,ELO3,EPT1,ERG2,FAT1,FLC1,FLC3,FOX2,GPI13,GPT2,GRE2,HFD1,HMG1,HMG2,IPT1,LCB4,LDB16,LPP1,LPX1,LSB6,MDH3,NMA111,NSG1,NSG2,NTE1,OAF1,OSH6,PCT1,PDH1,PGC1,PLB1,ROG1,RRI1,RSP5,SEI1,SKN1,TAZ1,TES1,TGL1,TGL2,TGL3,TGL4,TOR1,UPS2,YDR018C,YEH1,YEH2,YFT2,YJU3,YMR210W,YPC1,YPR147C,YSR3
mRNA processing (GO:0006397)	CUS2,DBR1,FKH1,PBP1,STO1
meiotic cell cycle (GO:0051321)	BMH1,BMH2,BNS1,CDA1,CDA2,CRR1,CSM2,CSM4,DIT1,DIT2,DMC1,EMI2,EXO1,FKS3,FPR3,GAC1,GAS4,GIP1,HOP1,HOP2,IME2,ISC10,LDS2,MAM1,MDS3,MEK1,MLH1,MSC3,MSC7,MSH5,NDJ1,NDT80,OSW2,PCH2,PMD1,QDR3,RAS2,RCK2,RDH54,RED1,RIM4,SHC1,SMK1,SPO11,SPO13,SPO19,SPO73,SPR1,SPR3,SPS18,SPS2,SPS4,SSP1,SUR7,TOR1,YNL194C,YOR338W,ZIP1,ZIP2
mitochondrial translation (GO:0032543)	FYV4,MBA1,MRPL50,MRPS17,RSM25
mitochondrion organization (GO:0007005)	ATG33,ATG41,BUL1,CAF4,COA6,CQD2,DCK1,FMP32,GEM1,HOT13,MBA1,MDM1,MDM36,MFB1,MIC12,MIC27,NGR1,OMA1,PBP1,PEP1,REX2,RSP5,SDH7,SED1,TAZ1,TPK3,UBP12,UBP2,UPS2,XDJ1,YDR381C-A,YNR040W
mitotic cell cycle (GO:0000278)	ACE2,ALK1,ALK2,AMN1,ARP10,AXL1,BEM2,BMH1,BNR1,BUD2,BUD4,BUD8,DMA2,EXO1,FIN1,FKH1,GAC1,HOS3,IGO2,KEL1,KIN4,MBP1,MLC2,MSA2,NPL4,NUR1,PPH22,PTK2,RAD30,RAX1,RAX2,SAP4,SFG1,SIS2,VHS1,VHS2,YBP2,YHP1,ZDS2
nucleobase-containing compound transport (GO:0015931)	BUL1,FLC1,FLC3,NEW1,NRT1,NUP120,POR2,RPS26A,RSP5,SAL1,YEA4,YMD8
nucleobase-containing small molecule metabolic process (GO:0055086)	ACS1,ADE16,ADE2,ADK2,BNA6,DCD1,ENO1,ENO2,FAA4,GLK1,HMG1,HNT2,HXK1,MTD1,NMA1,NMA2,PFK27,PGM2,PNC1,RNR3,SIS2,STD1,TYE7,URC2,URH1,VHS3,YNK1
nucleus organization (GO:0006997)	FUS2,NUP120,OSH3,PNC1,SYH1,ZDS2

oligosaccharide metabolic process (GO:0009311)	IMA5,MAL32,PGM2,SUC2,TPS3
organelle assembly (GO:0070925)	ATG23,ATG41,BLM10,DMA2,PBP1,PPH22,PUB1,RAS2,SAM1,SEI1,TPK1,TPK2,TPK3,YLR173W
organelle fission (GO:0048285)	AMN1,BMH1,BUD2,CAF4,CSM2,CSM4,DMA2,DMC1,FIN1,FPR3,GAC1,HOP1,HOP2,IME2,KEL1,KIN4,MAM1,MDM36,MEK1,MLH1,MSC3,MSC7,MSH5,NDJ1,NUR1,PCH2,RAD30,RCK2,RDH54,RED1,RIM4,SPO11,SPO13,ZIP1,ZIP2
organelle inheritance (GO:0048308)	BUL1,GEM1,MDM1,MDM36,MYO4
protein acylation (GO:0043543)	ACS1,EAF6,ERF2,HFI1,HPA2
protein alkylation (GO:0008213)	CTM1,FPR4,RKM1,SET5
protein folding (GO:0006457)	ACL4,AHA1,CAJ1,CUR1,EMC5,ERJ5,EUG1,FLC1,FPR1,MPD1,SBA1,XDJ1
protein glycosylation (GO:0006486)	KTR3,KTR4,KTR6,KTR7,MNT2,MNT3,PMT5,PMT6
protein lipidation (GO:0006497)	ERF2,GPI13
protein modification by small protein conjugation or removal (GO:0070647)	APJ1,BMH1,BMH2,BUL1,CSN9,DCN1,DMA2,LAG2,OTU1,RRI1,RRI2,RSP5,RUP1,SNT2,TUL1,UBP12,UBP16,UBP2,UBX3,WSS1,YPL191C,ZIP1,ZIP2
protein phosphorylation (GO:0006468)	ALK1,ALK2,CKA1,CMK2,HAL5,HRK1,IKS1,IME2,KIN1,KIN4,KSS1,LSP1,MAM1,MEK1,MKK2,NCE102,NNK1,POR2,PRR2,PSK2,PTC2,PTK2,RCK2,SAK1,SIP1,SKM1,SMK1,SSK22,TEL1,TOR1,TPK1,TPK2,TPK3,VHS1,YCK2,YCK3,YDL073W,YGK3,YPL150W
protein targeting (GO:0006605)	AST2,ATG23,ATG34,ATG41,BSD2,ERF2,FRT2,HOT13,MDH2,PEP1,RAS2,ROY1,SPL2,WSC4,XDJ1,YKR051W
proteolysis involved in cellular protein catabolic process (GO:0051603)	AMN1,ATE1,BLM10,BSD2,COS5,DER1,DFM1,DMA2,FAT1,GGA1,GGA2,HRT3,MFB1,MNL1,NMA111,NPL4,OMA1,PNG1,RPN14,RQC1,RSP5,TUL1,UBP2,UBX5,UCC1,YPF1
pseudohyphal growth (GO:0007124)	BMH1,BMH2,BUD8,DBR1,DIA1,DIA3,DNF3,ECM23,FKH1,FLO11,HMS1,ITR1,MDS3,NRG2,OSH3,RAS2,SAK1,SFG1,SIP1
rRNA processing (GO:0006364)	PUS7,REX2,RPL35A,RPS26A,RPS27B,RSP5,TSR3
regulation of cell cycle (GO:0051726)	ALK1,ALK2,AMN1,BEM2,BMH1,BMH2,BUD2,CSM4,DMA2,EXO1,FIN1,FKH1,FPR3,GAC1,HOP1,IGO2,IME2,KEL1,KIN4,KSS1,MDS3,MEK1,NDJ1,NUR1,PCH2,PTC2,RCK2,RED1,SIS2,SPO13,TOR1,YPR015C,ZDS2,ZIP1
regulation of organelle organization (GO:0033043)	AMN1,BNR1,DMA2,ENO1,ENO2,EST1,FIN1,FKH1,FPR3,GAC1,GEM1,HOP1,IME2,KEL1,LSB1,MEK1,MHP1,NDJ1,PBP1,PCH2,PIN3,PPH22,RAS2,RCK2,RED1,RSP5,SAM1,SBA1,SPO13,TDA2,TPK1,TPK2,TPK3,UBP12,UBP2,YGL015C,YGR042W,ZIP1
regulation of protein modification process (GO:0031399)	BMH1,BMH2,DCN1,FPR4,GIP1,GIP4,GRX3,GRX4,IGO2,LAG2,LSP1,MAM1,NCE102,POR2,PTC2,REG2,RUP1,SIS2,TOR1,XBP1,YDL073W,ZIP1,ZIP2
regulation of translation (GO:0006417)	ANB1,CAM1,DCS2,DXO1,ECM32,EFT1,EFT2,GUF1,IGO2,JSN1,MBA1,MTQ1,NCL1,NEW1,NGR1,PBP1,PPH22,PSK2,PUF2,RRT2,SLH1,TMA108,TPA1,TPK2,TPK3,TRF5,WHI4
regulation of transport (GO:0051049)	AGP2,ALY2,DFM1,GYL1,HAL5,KEL1,KIN1,MSG5,NCE102,PHO91,RSP5,YHL008C
response to chemical (GO:0042221)	AFB1,AGA2,ARR1,ATF2,AYT1,CAT8,CCP1,CIN5,CRS5,CRZ1,CSN9,CST6,CUP2,DAK1,DAK2,DDR2,DER1,DFM1,DNF1,DNF3,ECM5,FLR1,FRT2,GAL1,GPX1,GRX3,GRX4,GRX7,HAL5,HRT3,KIN1,KSS1,MF(ALPHA)2,MIG1,MIG2,MNL1,MSB2,MSG5,MSN2,NCL1,NMA111,NPL4,NQM1,NRG2,OAF1,OXR1,PDR10,PNG1,POG1,PRR2,PSR1,PSR2,PTC2,

	PTP3,RAS2,RCK2,RRI1,RRI2,RSP5,SCO2,SIP18,SLI1,SNT2,SRX1,SSK22,STB3,STB5,TMA19,TOR1,TRX3,UCC1,UGA2,XBP1,YCF1,YCK2,YCR102C,YDL073W,YDL124W,YGK3,YGL039W,YHB1,YHI9,YHK8,YJL055W,YKL071W,YRR1
response to heat (GO:0009408)	AHA1,CUR1,DDR2,GAC1,LSP1,MSN2,NMA111,NUP120,PSR1,PSR2,TOR1,WSC4,YGK3
response to osmotic stress (GO:0006970)	CIN5,FRT2,GPP2,HAL1,MSB2,NRG2,PSR1,PSR2,RCK2,SIS2,SSK22,STD1,STO1,YDL073W,YGK3,YGR066C
response to oxidative stress (GO:0006979)	CCP1,DDR2,ECM5,GPX1,GRX3,GRX4,GRX7,MSN2,NCL1,NQM1,OXR1,RCK2,SCO2,SNT2,SRX1,STB5,TMA19,TOR1,TRX3,UGA2,XBP1,YDL124W,YHB1,YRR1
response to starvation (GO:0042594)	DCS2,DDR2,DPL1,MIG1,MIG2,MSN2,PHO5,TAX4,TMT1,YGR066C,YLR173W,YPF1
ribosomal large subunit biogenesis (GO:0042273)	ACL4,REH1,RPL35A,RPS26A,SSF2
ribosomal small subunit biogenesis (GO:0042274)	NEW1,RPS27B,TSR3
ribosome assembly (GO:0042255)	RPS26A,RPS27B,SSF2
sno(s)RNA processing (GO:0043144)	TRF5
sporulation (GO:0043934)	BMH1,BMH2,CDA1,CDA2,CRR1,CTS2,DIT1,DIT2,EMI2,FKS3,GAS4,GIP1,ISC10,LDS2,MDS3,OSW2,PMD1,PTP3,QDR3,RAS2,RIM4,SHC1,SMK1,SPO19,SPO73,SPR1,SPR3,SPS18,SPS2,SPS4,SSP1,SUR7,YNL194C,YOR338W
tRNA processing (GO:0008033)	ABP140,AIR2,CBR1,ISU1,NCL1,PPM2,PUS1,PUS7,RSP5,RTT10,TAN1,TRM1,TRM11,TRM732,TRM8,TYW1
telomere organization (GO:0032200)	ESC1,EST1,EST3,EXO1,HEK2,NUP120,PNC1,SBA1,SET5,TEL1,YGR042W,YKU70,YKU80
transcription by RNA polymerase I (GO:0006360)	CKA1,ISW1,TOR1
transcription by RNA polymerase II (GO:0006366)	ACE2,ARR1,BMH1,CAF16,CAF4,CAM1,CAT8,CIN5,CRZ1,CST6,CUP2,EMI2,FKH1,FLO8,FPR1,GAL80,HAL1,HFI1,HOS3,ISW1,JHD1,MED2,MET28,MIG1,MIG2,MSN2,MSS11,NDT80,NRG2,NUP120,OAF1,POG1,PRR2,PSR1,PSR2,ROX3,RSF1,RSP5,SKM1,SMP1,SNT2,STB3,STB5,STD1,TOD6,TYE7,XBP1,YHP1,YOR338W,YRM1,YRR1
transcription by RNA polymerase III (GO:0006383)	CKA1
translational initiation (GO:0006413)	TIF3,TOR1
transposition (GO:0032196)	DBR1,YCL074W
vacuole organization (GO:0007033)	ATG23,ATG41,ENO1,ENO2,IVY1,NCE102,PPH22,VTC1,VTC2,VTC3,YCF1,YHR138C,YLR173W
vesicle organization (GO:0016050)	ATG15,BLI1,BLS1,SVP26
vitamin metabolic process (GO:0006766)	ABZ2,BIO5,ECM31,FMS1,PAN6,PHO3
